# Altered Local and Large-Scale Dynamic Functional Connectivity Variability in Posttraumatic Stress Disorder: A Resting-State fMRI Study

**DOI:** 10.3389/fpsyt.2019.00234

**Published:** 2019-04-12

**Authors:** Shishun Fu, Xiaofen Ma, Yunfan Wu, Zhigang Bai, Yin Yi, Mengchen Liu, Zhihong Lan, Kelei Hua, Shumei Huang, Meng Li, Guihua Jiang

**Affiliations:** ^1^The Second School of Clinical Medicine, Southern Medical University, Guangzhou, China; ^2^The Department of Medical Imaging of Guangdong Second Provincial General Hospital, Guangzhou, China; ^3^The Department of Medical Imaging of Affiliated Hospital, Inner Mongolia University for Nationalities, Hohhot, China; ^4^Guangdong Medical University, Dongguan, China

**Keywords:** posttraumatic stress disorder, resting-state functional magnetic resonance imaging, dynamic functional connectivity, regional homogeneity, default mode network

## Abstract

Posttraumatic stress disorder (PTSD) is a psychiatric condition that can emerge after exposure to an exceedingly traumatic event. Previous neuroimaging studies have indicated that PTSD is characterized by aberrant resting-state functional connectivity (FC). However, few existing studies on PTSD have examined dynamic changes in resting-state FC related to network formation, interaction, and dissolution over time. In this study, we compared the dynamic resting-state local and large-scale FC between PTSD patients (*n* = 22) and healthy controls (HC; *n* = 22; conducted as standard deviation in resting-state local and large-scale FC over a series of sliding windows). Local dynamic FC was examined by calculating the dynamic regional homogeneity (dReHo), and large-scale dynamic FC (dFC) was investigated between regions with significant dReHo group differences. For the PTSD patients, we also investigated the relationship between symptom severity and dFC/dReHo. Our results showed that PTSD patients were characterized by I) increased dynamic (more variable) dReHo in left precuneus (PCu); II) increased dynamic (more variable) dFC between the left PCu and left insula; and III) decreased dFC between left PCu and left inferior parietal lobe (IPL), and decreased dFC between left PCu and right PCu. However, there is no significant correlation between the clinical indicators and dReHo/dFC after the family-wise-error (FWE) correction. These findings provided the initial evidence that PTSD is characterized by aberrant patterns of fluctuating communication within brain system such as the default mode network (DMN) and among different brain systems such as the salience network and the DMN.

## Introduction

Posttraumatic stress disorder (PTSD) is a psychiatric condition that can emerge after exposure to an exceedingly traumatic event (1). In the general population, PTSD occurs most commonly after traffic accidents and affects 10%–32% of those involved within 12 months after the event (2). Symptoms of PTSD include intrusive memories, hypervigilance, insomnia, and emotional numbing (3). Previous studies have indicated that PTSD patients exhibited abnormal interactions among the brain systems (4, 5). For example, Zhang et al. found that the dorsolateral prefrontal cortex showed increased resting-state functional connectivity (FC) with the visual cortex, suggesting that the disrupted frontal-occipital system may be associated with the dysfunction of visual information processing (5). One of robustly identifiable networks is the default mode network (DMN) (6), which is involved in processing self-relevant stimuli (7, 8). The dysfunction of the DMN in PTSD patients may indicate impaired self-generated thoughts and autobiographical memory during rest (9).

One effective approach for exploring brain communication is through the analysis of resting-state fMRI studies (10). Recent resting-state studies in both animals and humans have revealed the dynamic nature of the spatiotemporal organization of blood oxygen level-dependent (BOLD) signals (11–13). Due to unconstrained mental activity, the resting state even shows more dynamic features than in task-stimuli studies (14). A recent study of dynamic FC network indicated that the static FC represented average connectivity across different dynamic states during the whole scanning period; it may not be sensitive enough to detect the alteration of neurofluctuations (15). In order to investigate the dynamic features of inter-regional BOLD signal fluctuations over temporal scales, the sliding window analysis of dynamic FC (dFC) was developed. This approach measured the variety correlations among discrete (large-scale) brain regions (16) using a short, sliding temporal window. Kaiser et al. found that the resting-state dFC revealed the interactions among networks or subnetworks over time (17). Early studies suggested that the dFC can be associated with the changes in arousal (18) and vigilance (19) since hypervigilance and hyper-arousal are two typical symptoms of PTSD. We proposed to use dFC to investigate the characteristic features of PTSD in the resting state. Moreover, since changes in brain network topology are associated with those in local brain activity (20), it was reasonable for us to measure both the large-scale and local dynamic FC in our study.

Regional homogeneity (ReHo) is one of the commonly used algorithms in measuring local FC (21–23). ReHo is a reliable measurement technique and robust against noise in the fast imaging sequence data (24). A prior animal study has suggested an association between ReHo variability and different states of neural activity (25). A recent study of dReHo using the sliding-window approach also indicated that brain regions with high dReHo fluctuation tended to be functional hubs in brain systems (26). A resting-state study has shown that the gene variants affected dReHo in attention-deficit/hyperactivity disorder (27). These findings introduced the clinical potential of dReHo analysis.

The investigation of dFC and dReHo in the resting state may provide new insight into the aberrant brain connectivity in PTSD. Previous studies investigating major depression (28), schizophrenia (29, 30), and bipolar disorder (31) showed abnormal dFC and dReHo under the resting state of these patients, and all of these psychiatry researches found aberrant dFC or aberrant dynamic local activities in the DMN. A possible explanation for these abnormalities is the dynamic nature of the DMN, which exhibits dynamic interactions with a number of other brain systems in the resting state (32). Kaiser et al. (17) indicated that the investigation of altered dynamic activity in areas of the DMN may be important in understanding the pathophysiology of psychiatric disorders.

Although there are few studies available that have focused on the dynamic brain activity in PTSD, prior static studies have suggested that the symptoms of PTSD are associated with the DMN. Mounting evidence has indicated that PTSD is associated with aberrant DMN connectivity (33–35). A static FC (sFC) study suggested that the aberrant activities in the DMN can be a predictor of the symptom severity of PTSD (8). A previous study also approved that the static fMRI data can be used to discriminate the PTSD from HC by using the multilevel parametric classification approach (36). A recent study compared the accuracy of sFC to the accuracy of dFC in classifying PTSD patients and HC (37). The results showed that the peak classification accuracy of dFC reached 94.2%, while the peak classification accuracy of sFC was 86.7%; this research indicated that the temporal dFC is a better predictor than sFC of the diagnostic features of PTSD. Additionally, this study indicated that, in comparison with the HC, the PTSD patients were characterized by decreased temporal variability of brain connectivity. Preti et al. also indicated that PTSD patients often stay trapped in one state and exhibited a decreased dFC in comparison with HC subjects (38). All of these studies indicated that the aberrant connectivity variability of brain networks is vital in the investigation of the neurophysiological mechanism of PTSD.

In order to explore the characteristic resting-state temporal variability of PTSD, we decided to measure both large-scale and local dynamic FC. Based on previous findings, we hypothesized that PTSD patients would exhibit altered dReHo in regions within the DMN. We also expected regions with dReHo alterations to show aberrant dFC and the connectivity measures to be associated with subjects’ symptomatology.

## Method

### Subjects

Permission to undertake this study was granted by the ethics committee of Guangdong Second Provincial General Hospital. In January and February 2017, we recruited 30 trauma-exposed subjects from a serious highway traffic accident in Guangdong province. Prior to the examination, none of the patients had undergone any psychotherapy. The inclusion criteria for the PTSD patients were as follows: I) age >18 years; II) right-hand dominance; III) no preexisting psychiatric disorders or physical conditions as determined by a structural clinical interview using the *Diagnostic and Statistical Manual of Mental Disorders*, 4th edition (DSM-IV); IV) no psychiatric medications or substance abuse; V) no MR imaging contraindications; VI) no head trauma or neurologic disorders; VII) fulfills the criteria of DSM-IV and has a Clinical-Administered PTSD Scale (CAPS) score >40; and VIII) not pregnant or nursing. After considering the strict requirements, eight subjects were excluded, five of them for failing to obtain the CAPS score >40. Twenty-two demographically matched healthy controls (HCs) were recruited for this study. The inclusion criteria for HCs were as follows: I) age >18 years; II) right-hand dominance; III) no preexisting psychiatric disorders or physical conditions as determined by a structural clinical interview using the DSM-IV; IV) no psychiatric medications or substance abuse; V) no MR imaging contraindications; and VI) not pregnant or nursing. Each participant provided written informed consent, which was obtained prior to the MRI scanning.

### Assessment of Mental Status

PTSD diagnosis was determined following the DSM-IV diagnostic criteria. Before undergoing resting-state MRI, all PTSD patients were screened with CAPS (39) in order to estimate the intensity and frequency of the symptoms. In addition, emotion assessments were conducted of all participants, including the Self-rating Anxiety Scale (SAS) (40) and the Self-rating Depression (SDS) (41), in order to evaluate the emotional status. A further Structured Clinical Interview for DSM-IV was also performed to evaluate psychiatric disorder comorbidities.

### Magnetic Resonance Imaging Data Acquisition

Each of the participants underwent a resting-state MRI in a 3.0-T MR imager (Ingenia; Philips, Best, The Netherlands) equipped with a 32-channel head coil at the Department of Medical Imaging in Guangdong Second Provincial General Hospital. A diagnostic T1-weighted image and a T2 fluid attenuated inversion recovery (T2-FLAIR) image were taken to exclude participants with brain lesions. The resting-state fMRI data were acquired using gradient echo-planar imaging (EPI) with the following parameters: repetition time (TR)/echo time (TE) = 2,000 ms/30 ms; matrix = 64 × 64; field-of-view = 230 mm × 230 mm; flip angle = 90; slice thickness = 3.6 mm, 0.6-mm gap; interleaved scanning; 38 transverse slices covering the whole brain at all 240 volumes were acquired for each participant within 480 s; each volume was aligned along the anterior–posterior commissure. Each participant was instructed to lie still and to avoid falling asleep or thinking of anything in particular during MR scanning.

### Resting-State Functional Magnetic Resonance Imaging Data Preprocess

Standard preprocessing of the functional images was performed with the DPARSF 4.3 Advanced Edition (http://rfmri.org/DPARSF) and the SPM12 package (www.fil.ion.ucl.ac.uk/spm) based on MATLAB (Mathworks, Inc., Natick, MA, USA). The first 10 volumes of each dataset were discarded for signal equilibration. The remaining data were performed using slice timing correction and realignment and co-registered with the anatomical scan. The co-registered T1-weighted images were segmented into gray matter, white matter, and cerebrospinal fluid. And then the functional images were normalized into the Montreal Neurological Institute (MNI) space with a voxel size of 3 × 3 × 3 mm^3^. The head movement parameters were obtained from the realignment steps in the DPARSF. We took the mean FD Jenkinson (42) as the head motion reference standard. We eliminated the subjects with motion (mean FD Jenkinson) greater than 2 × standard deviation (SD) above the group mean motion as recommended in a previous study (43). No subject was eliminated in this step. There was no significant difference in head motion between the PTSD patients and the HC (see **Table 1**). Linear detrending processing was conducted to remove the linear signal drift. Individual-level regression analysis was conducted to minimize the influence of head motion (Friston 24 model), white matter signal noise, and cerebrospinal fluid signal noise. A temporal band-pass filter (0.0167–0.10 Hz) was applied to the data to remove the physical noise and any frequencies for which the period was shorter than that of a single sliding window (44). We performed spatial smoothing with a 6-mm full-width at half-maximum (FWHM) kernel before performing the dReHo group analysis. As for the dFC, we performed the spatial smoothing with a 6-mm FWHM kernel before the linear detrending and nuisance signals regression, and band-pass filtering. Considering the size of FWHM Gaussian kernel might affect the results of dReHo/dFC analysis (45, 46), we used 4- and 8-mm FWHM Gaussian kernel to test the consistency of our results (45, 46) (see **Supplementary Figures 1** and **2**).

**Table 1:** Demographic and clinical data.

Characteristic	PTSD (*n* = 22)	HC (*n* = 22)	*t* value	*P* value
Age (years)	37.36 ± 8.95	40.32 ± 10.34	−1.014	0.317
Gender (M/F)	8/14	8/14		
Head motion	0.169 ± 0.443	0.159 ± 0.441	0.073	0.942
Education (years)	11.82 ± 3.22	10.45 ± 4.25	1.200	0.237
CAPS	51.45 ± 6.93			
SAS	36.09 ± 8.11	38.18 ± 6.02	−0.971	0.337
SDS	38.05 ± 9.49	39.09 ± 8.08	−0.393	0.696

Demographic data are presented as mean ± SD. PTSD, posttraumatic stress disorder; HC, healthy control; CAPS, Clinical-Administrated PTSD Scale; SAS, Self-rating Anxiety Scale; SDS, Self-rating Depression Scale. The P-value was obtained by the two-sample t test.

### Computation of dReHo and dFC

ReHo calculation: The ReHo algorithm measures voxel-wise short-distance FC with Kendall’s coefficient of concordance (23) using the following formula:

$$W=\frac{\sum_{i=1}^{N}R_{i}^{2}-N{\bar{R}}^{2}}{\frac{1}{12}K^{2}(N^{3}-N)}$$

where *W* is the Kendall’s coefficient of concordance among the given voxels, *N* denotes the length of the time series, *K* = 27 is the size of the voxel cluster containing 3 × 3 × 3 adjacent voxels, *R* *_i_* denotes the summation of the rankings of the BOLD signal amplitude of all *K* voxels at the *i*th time point, and *R* is the mean of *R* *_i_*.

To compute the dReHo for these data, the time course was segmented into 60-s Hamming windows (30 dynamics). By sliding the onset of each window by 2 dynamics (4 s), for a total of 101 overlapping windows in the first level analysis, the dReHo was estimated by using the calculated SD of the ReHo through the windows at each voxel, yielding a set of ReHo maps for each participant.

Two-sample *t*-test with head motion parameters (mean FD Jenkinson values), age, and sex as covariates was performed to test the difference in dReHo maps between the PTSD patients and HC at each voxel. Multiple comparisons correction was performed with Gaussian random field (GRF) theory at the cluster level (minimum *z* > 3.54; cluster significance: *p* < 0.05, two-tailed GRF corrected).

To determine whether the dReHo metrics were associated with clinical indicators, we performed general linear models with the clinical indicators (CAPS, SAS, SDS) and mean dReHo values from clusters with significant group differences as independent variables, and head motion parameters, age, and sex as covariates. The correlation analysis was accomplished with the SPSS software with a significance threshold of *p* < 0.05 (uncorrected).

Voxel-wise seed-based FC analyses were performed using the DPARSF 4.3. We employed the aberrant dReHo region, which we calculated above, as a seed region. Then we used the sliding-window approach as we have used in the dReHo calculation; the time course was segmented into 60-s Hamming windows by sliding the onset of each window by 2 dynamics, for a total of 101 overlapping windows in the first level analysis. Within each sliding window, the whole brain FC maps for the seed region were computed as the Fisher *z* transformed Pearson correlation coefficient between the averaged time course of all voxels in the seed and the time course of all other voxels in the whole brain, yielding a set of sliding window *z*FC maps for each participant. The dFC was estimated by calculating the standard deviation in *z*FC values through windows at each voxel.

Two-sample *t*-test with head motion parameters (mean FD Jenkinson values), age, and sex as covariates was performed to investigate the difference of dFC values between the PTSD group and the HC group at each voxel. Multiple comparisons were performed with GRF correction at the cluster level (minimum *z* > 3.29; cluster significance: *p* < 0.05, two-tailed GRF corrected).

To explore the relationship between dFC metrics and clinical indicators, we performed general linear models with the clinical indicators and mean dFC values from clusters with significant group differences as independent variables, and head motion parameters, age, and sex as covariates. The correlation analysis was accomplished with SPSS software with significance threshold of *p* < 0.05 (uncorrected).

## Results

**Figure 1:**
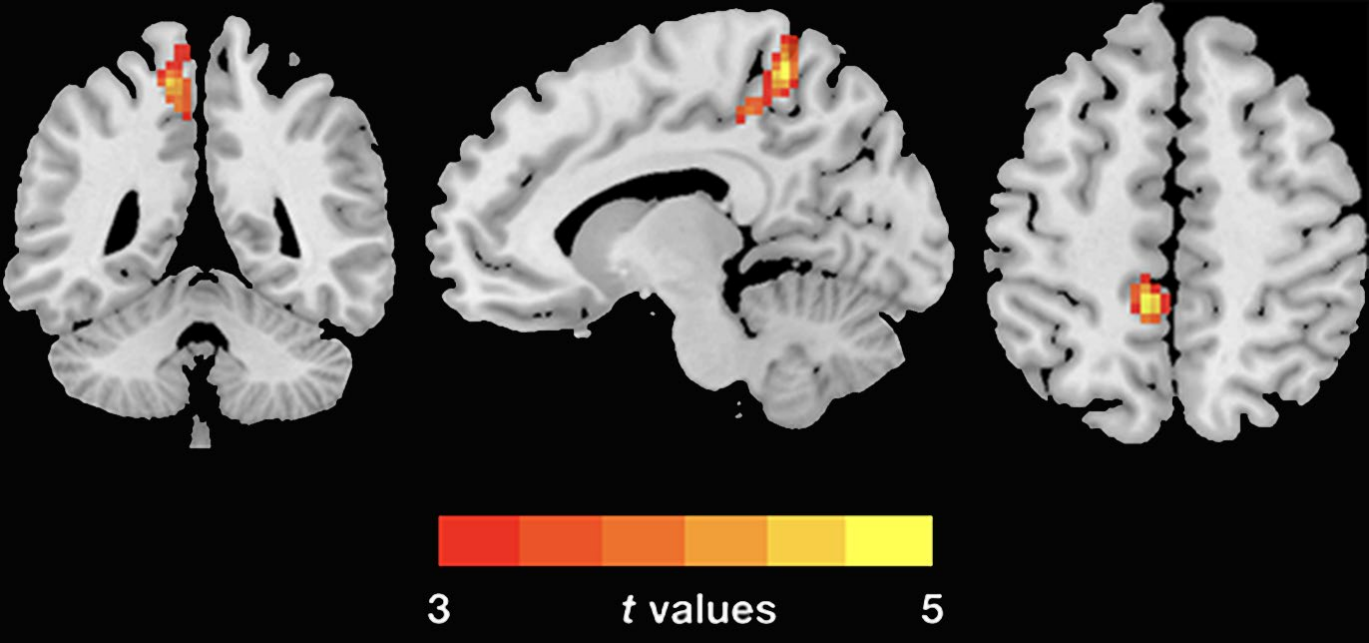
Group differences of dReHo variability were revealed by two-sample *t*-test. The PTSD group shows increased (warm color) dReHo variability in the left PCu (cluster size: 104; AAL: Precuneus_L; Brodmann area 7; MNI coordinates: X: −12 Y: −48 Z: 60; peak *t*-value = 5.1868) relative to the HC. The Gaussian random theory was used for cluster-level multiple comparison correction (minimum *z* > 3.54; cluster significance *p* < 0.05, GRF corrected). dReHo, dynamic regional homogeneity; PTSD, posttraumatic stress disorder; AAL, anatomical automatic labeling; PCu, precuneus; MNI, Montreal Neurological Institute.

**Figure 2:**
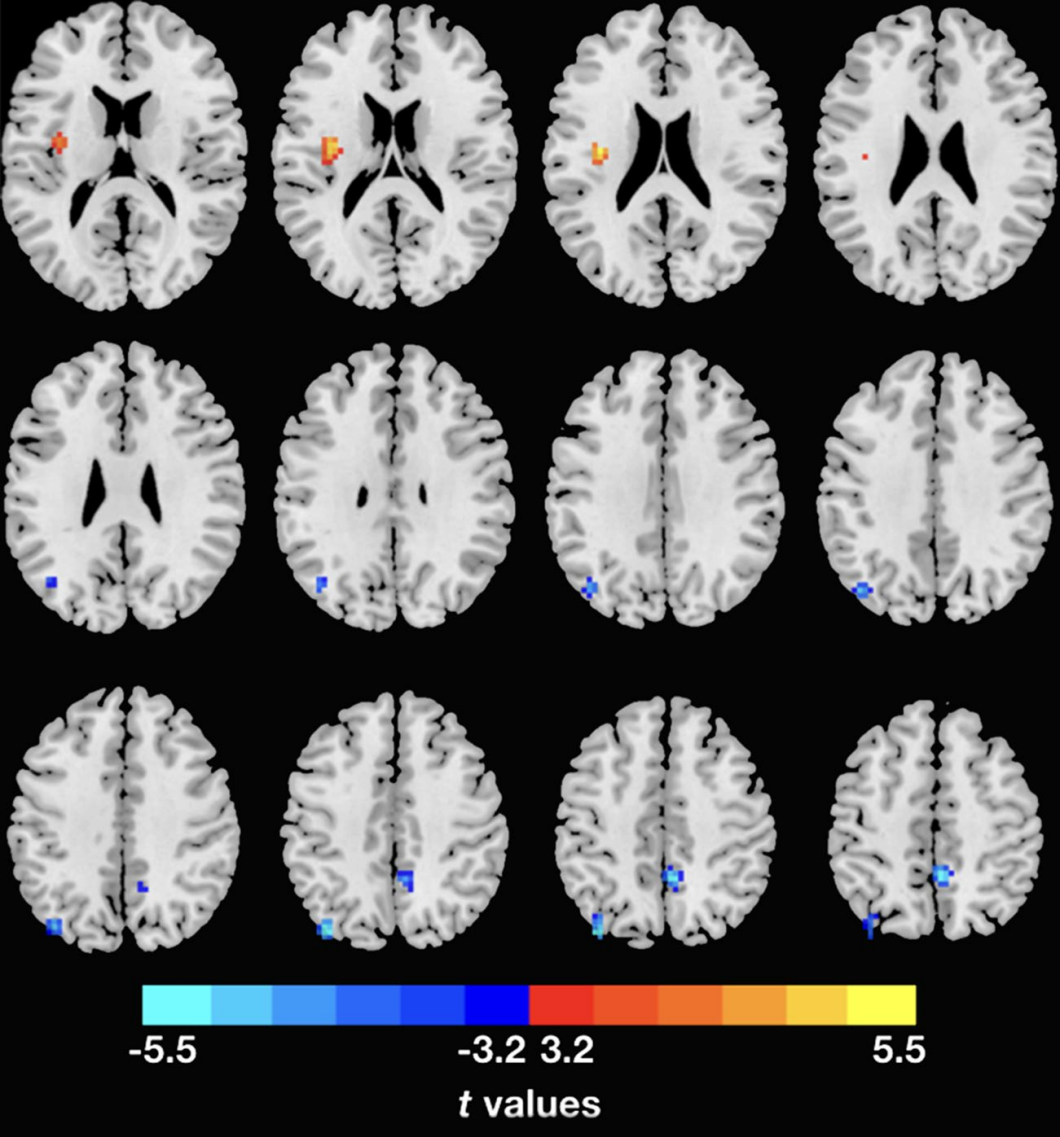
Inter-regional dFC differences with regions showing significant dReHo differences between groups. The PTSD group shows increased dFC variability in left insula, but decreased dFC variability in right PCu and left IPL relative to the HC. Gaussian random theory was used for cluster-level multiple comparison correction (minimum *z* > 3.29; cluster significance *p* < 0.05, GRF corrected). dFC, dynamic functional connectivity; dReHo, dynamic regional homogeneity; PTSD, posttraumatic stress disorder; PCu, precuneus; IPL, inferior parietal lobe.

The demographic and clinical data are summarized in **Table 1**. Inconsistent with our prediction, compared with the HC, the PTSD patients exhibited an increased dReHo (more variability) in the left posterior cingulate cortex (PCC)/precuneus (PCu) (**Figure 1**). We also found a decreased dFC (less variability) between the left and right PCu, and the left inferior parietal lobe (IPL)/angular gyrus (AG), but increased dFC between the PCC and the left insula (**Table 2** and **Figure 2**).

**Table 2:** Comparison of dFC between PTSD and HC.

Brain region	Cluster size	MNI coordinates			AAL	Brodmann’s area	Peak *t* value
		X	Y	Z			
R PCu	44	3	−48	45	Precuneus_R	7	−4.0992
L IPL	62	−38	−78	42	Parietal_Inf_L	19	−4.1411
L Insula	33	−36	−12	21	Insula_L	13	4.6200

L, left; R, right; MNI, Montreal Neurological Institute; dFC, dynamic functional connectivity; AAL, anatomical automatic labeling; PCu, precuneus; IPL, inferior parietal lobe.

Next, we conducted analyses to test the association between the clinical indicators (CAPS, SAS, SDS) of PTSD and dReHo within the PTSD group. There is no significant correlation between the clinical indicators and dReHo/dFC after FWE correction (see **Supplementary Figure 4**).

## Discussion

In this study, by using resting-state dReHo analysis, we determined the local aberrant variability in the left PCu. The PCu is a key hub in the DMN of human brain (47). Aberrant resting-state temporal dynamic brain activities were found in the dReHo and large-scale dFC of specific brain regions, which were mainly located in the posterior DMN (pDMN) and the primary region of the salience network (SN). These observations provide new insights into the aberrant brain activities in PTSD.

This study revealed a significantly increased dReHo (more variability) in left PCu, suggesting that the neurofluctuation of the left PCu is unstable in the PTSD patients in the resting state. The PCu is a key hub of the pDMN (47–49) and is considered to be involved in self-referential processing (7) and autobiographic memory (50). In the resting state, the DMN exhibits dynamic interaction with a number of brain systems, such as the frontal–parietal control network and the dorsal attention network (32). Although we found an aberrant local connectivity variety in the PCu, the evidence of aberrant DMN connectivity in PTSD patients is not entirely persuasive. In order to explore the dynamic interactions among the DMN and other brain networks, we employed the left PCu as the seed region and carried out the dFC of the whole brain.

Several studies have identified aberrant activities in the DMN of PTSD patients (6, 34, 35). In the present study, using seed-based dFC, we identified lower-variability regions located in the right PCu and the left IPL. These results suggest that compared with the normal controls, the PTSD group exhibits decreased dFC (less variability) within the pDMN. We suspected that this restrained neurofluctuation within the pDMN represents decreased regulation of the self-referential processing. Previous studies using the independent component analysis identified the aberrant pDMN in PTSD patients (4, 51). Furthermore, Zhang et al. found a decreased intranetwork connectivity within the pDMN by measuring resting-state sFC. These researches suggested that the decreased FC in pDMN was associated with the dysfunction of evaluation of the self-related events in PTSD. The interaction between DMN and the executive control systems, which includes dorsal lateral middle frontal cortex and the IPL (52), is essential in regulating the self-generated thought (32). In the present study, a deceased dFC was found between the left PCu and the left IPL. Therefore, we inferred that the deceased dFC suggested a discrete FC state between the DMN and the executive control system, which may induce the dysregulation of self-referential processing. Previous studies indicated that the IPL is engaged in mediating visuospatial processing (53), which is critical when dealing with life-threatening events (54). Additionally, previous study indicated that the IPL is a vulnerable brain region to the neurotoxic effects of stress (55). Therefore, we suspected that the aberrant dFC between the PCu and the IPL might be a potential biomarker of PTSD.

In the present study, the left insula was the only region in the brain that exhibited significant positive dynamic correlation with the left PCu in the PTSD group. The insula is a key hub in the SN and is thought to be involved in the detection of personally salient internal and external stimuli that guide behaviors in order to maintain equilibrium (56). In addition, the insula is thought to be involved in mediation of the “switching” between activation of the DMN and the central executive network (CEN) to direct appropriate behavioral responses to the salience stimuli (57). Therefore, we suspected that the positive dynamic correlation between the left PCu and left insula might suggest an excessive interaction between the SN and the DMN. Previous studies using the graph theory approach identified dysregulation in three intrinsic brain networks (1, 58). Lei et al. found a disequilibrium among the CEN, DMN, and SN and suggested that the SN was crucial to the PTSD symptoms (58). Previous resting-state sFC study also revealed an increased correlation between the DMN seed region [PCC and ventromedial prefrontal cortex (vmPFC)] and SN (insula and precentral sulcus) (59). They found a positive correlation between the PTSD symptom severity and the vmPFC-precentral sulcus FC values. Our results provided an additional piece of evidence that, compared with the HC, the PTSD patients exhibited more variable connectivity between the DMN and the SN.

There are some limitations in the present study that should be highlighted. Firstly, little information is available on the meaning of the resting-state dFC in neurocognitive functioning. For example, it remains unknown whether the abnormal dynamic activities in the resting state are intrinsic properties or are affected by the present-moment cognitive activities (17, 60). As the number of resting-state dFC studies grows, we may gain a better understanding of these properties and their relation to the psychopathology. Secondly, since the dFC based on the sliding-window approach is composed of a few time points, the dynamic analysis is particularly sensitive to the physiological noise (61). Although, we did not denoised the physiological noise individually, we denoised the physiological noise in the preprocessing steps and group-level test and we also chose a relatively large window size in order to diminish the adverse effects of physiological noise. Thirdly, we only examined significant differences in regions exhibiting abnormal dynamic activity to focusing on the dynamic pattern related to PTSD; further exploration of static results is needed in our future works. Fourthly, the correlation results did not survive the FWE correction, so further exploration of the abnormal dynamic patterns and CAPS subscales is needed in our future large sample research to evaluate the relations between the dynamic patterns and specific clinical symptoms severity, such as intrusive memory and flash back.

In conclusion, this resting-state dFC (combine the dReHo and dFC) study provided evidence that the PTSD patients exhibited aberrant dReHo and dFC in comparison with the HC. Decreased variability within the DMN may suggest dysfunction of self-referential processing in PTSD patients, while increased variability between the insula and PCu may suggest dysregulation between the DMN and the SN.

## Ethics Statement

Permission to undertake this study was granted by the ethics committee of Guangdong Second Provincial General Hospital.

## Author Contributions

SF designed the experiment. SF and XM carried out the experiment. ZB collected and sorted out the data. YW, MLiu, YY, ZL, SH, MLi, and KH helped on data management and processing. SF and GJ wrote the manuscript.

## Conflict of Interest Statement

The authors declare that the research was conducted in the absence of any commercial or financial relationships that could be construed as a potential conflict of interest.

## References

[1] Yehuda R Hoge CW McFarlane AC Vermetten E Lanius RA Nievergelt CM Post-traumatic stress disorder. Nat Rev Dis Primers (2015) 1:15057. 10.1038/nrdp.2015.57 27189040

[2] YaŞan A Güzel A Tamam Y Ozkan M Predictive factors for acute stress disorder and posttraumatic stress disorder after motor vehicle accidents. Psychopathology (2009) 42:236–41. 10.1159/000218521 19451756

[3] American Psychiatric Publishing, Inc DSM-IV-TR: Diagnostic and statistical manual of mental disorders, text revision. Washington (2000).

[4] Ke J Zhang L Qi R Xu Q Zhong Y Liu T Typhoon-related post-traumatic stress disorder and trauma might lead to functional integration abnormalities in intra- and inter-resting state networks: a resting-state fMRI independent component analysis. Cell Physiol Biochem (2018) 48:99–110. 10.1159/000491666 30001548

[5] Zhang Y Xie B Chen H Li M Liu F Chen H Abnormal functional connectivity density in post-traumatic stress disorder. Brain Topogr (2016) 29:1–7. 10.1007/s10548-016-0472-8 26830769

[6] Akiki TJ Averill CL Wrocklage KM Scott JC Averill LA Schweinsburg B Default mode network abnormalities in posttraumatic stress disorder: a novel network-restricted topology approach. NeuroImage (2018) 176:1–27. 10.1016/j.neuroimage.2018.05.005 29730491 PMC5976548

[7] Bluhm RL Williamson PC Osuch EA Frewen PA Stevens TK Boksman K Alterations in default network connectivity in posttraumatic stress disorder related to early-life trauma. J Psychiatry Neurosci (2009) 34:187–94. http://jpn.ca/vol34-issue3/34-3-187/PMC2674971 19448848

[8] Lanius RA Bluhm RL Coupland NJ Hegadoren KM Rowe B Théberge J Default mode network connectivity as a predictor of post-traumatic stress disorder symptom severity in acutely traumatized subjects. Acta Psychiatr Scand (2009) 121:33–40. 10.1111/j.1600-0447.2009.01391.x 19426163

[9] Koch SBJ van Zuiden M Nawijn L Frijling JL Veltman DJ Olff M Aberrant resting-state brain activity in posttraumatic stress disorder: a meta-analysis and systematic review. Depress Anxiety (2016) 33:592–605. 10.1002/da.22478 26918313

[10] Biswal BB Resting state fMRI: a personal history. NeuroImage (2012) 62:938–44. 10.1016/j.neuroimage.2012.01.090 PMC12911935 22326802

[11] Chang C Glover GH Time–frequency dynamics of resting-state brain connectivity measured with fMRI. NeuroImage (2010) 50:81–98. 10.1016/j.neuroimage.2009.12.011 20006716 PMC2827259

[12] Hutchison RM Womelsdorf T Gati JS Everling S Menon RS Resting-state networks show dynamic functional connectivity in awake humans and anesthetized macaques. Hum Brain Mapp (2012) 34:2154–77. 10.1002/hbm.22058 PMC6870538 22438275

[13] Keilholz SD Magnuson ME Pan W-J Willis M Thompson GJ Dynamic properties of functional connectivity in the rodent. Brain Connect (2013) 3:31–40. 10.1089/brain.2012.0115 23106103 PMC3621313

[14] Deco G Ponce-Alvarez A Mantini D Romani GL Hagmann P Corbetta M Resting-state functional connectivity emerges from structurally and dynamically shaped slow linear fluctuations. J Neurosci (2013) 33:11239–52. 10.1523/JNEUROSCI.1091-13.2013 PMC3718368 23825427

[15] Liu F Wang Y Li M Wang W Li R Zhang Z Dynamic functional network connectivity in idiopathic generalized epilepsy with generalized tonic-clonic seizure. Hum Brain Mapp (2017) 38:957–73. 10.1002/hbm.23430 PMC6866949 27726245

[16] Handwerker DA Roopchansingh V Gonzalez-Castillo J Bandettini PA Periodic changes in fMRI connectivity. NeuroImage (2012) 63:1712–9. 10.1016/j.neuroimage.2012.06.078 PMC4180175 22796990

[17] Kaiser RH Whitfield-Gabrieli S Dillon DG Goer F Beltzer M Minkel J Dynamic resting-state functional connectivity in major depression. Neuropsychopharmacology (2016) 41:1822–30. 10.1038/npp.2015.352 PMC4869051 26632990

[18] Chang C Metzger CD Glover GH Duyn JH Heinze H-J Walter M Association between heart rate variability and fluctuations in resting-state functional connectivity. NeuroImage (2013) 68:93–104. 10.1016/j.neuroimage.2012.11.038 23246859 PMC3746190

[19] Thompson GJ Magnuson ME Merritt MD Schwarb H Pan W-J McKinley A Short-time windows of correlation between large-scale functional brain networks predict vigilance intraindividually and interindividually. Hum Brain Mapp (2012) 34:3280–98. 10.1002/hbm.22140 PMC6870033 22736565

[20] Gross T Blasius B Adaptive coevolutionary networks: a review. J R Soc Interface (2008) 5:259–71. 10.1098/rsif.2007.1229 PMC2405905 17971320

[21] Guo W-B Liu F Xue Z-M Yu Y Ma C-Q Tan C-L Abnormal neural activities in first-episode, treatment-naïve, short-illness-duration, and treatment-response patients with major depressive disorder: a resting-state fMRI study. J Affect Disord (2011) 135:326–31. 10.1016/j.jad.2011.06.048 21782246

[22] Liu F Hu M Wang S Guo W Zhao J Li J Abnormal regional spontaneous neural activity in first-episode, treatment-naive patients with late-life depression: a resting-state fMRI study. Prog Neuropsychopharmacol Biol Psychiatry (2012) 39:326–31. 10.1016/j.pnpbp.2012.07.004 22796277

[23] Zang Y Jiang T Lu Y He Y Tian L Regional homogeneity approach to fMRI data analysis. NeuroImage (2004) 22:394–400. 10.1016/j.neuroimage.2003.12.030 15110032

[24] Zuo X-N Xu T Jiang L Yang Z Cao X-Y He Y Toward reliable characterization of functional homogeneity in the human brain: preprocessing, scan duration, imaging resolution and computational space. NeuroImage (2013) 65:374–86. 10.1016/j.neuroimage.2012.10.017 PMC3609711 23085497

[25] Hudetz AG Liu X Pillay S Dynamic repertoire of intrinsic brain states is reduced in propofol-induced unconsciousness. Brain Connect (2015) 5:10–22. 10.1089/brain.2014.0230 24702200 PMC4313411

[26] Deng L Sun J Cheng L Tong S Characterizing dynamic local functional connectivity in the human brain. Sci Rep (2016) 6:26976. 10.1038/srep26976 27231194 PMC4882585

[27] Kim JI Yoo JH Kim D Jeong B Kim B-N The effects of GRIN2B and DRD4 gene variants on local functional connectivity in attention-deficit/hyperactivity disorder. Brain Imaging Behav (2017) 12:247–57. 10.1007/s11682-017-9690-2 28258362

[28] Qiu L Xia M Cheng B Yuan L Kuang W Bi F Abnormal dynamic functional connectivity of amygdalar subregions in untreated patients with first-episode major depressive disorder. JPN (2018) 43:262–72. 10.1503/jpn.170112 PMC6019355 29947609

[29] Damaraju E Allen EA Belger A Ford JM McEwen S Mathalon DH Dynamic functional connectivity analysis reveals transient states of dysconnectivity in schizophrenia. Neuroimage Clin (2014) 5:298–308. 10.1016/j.nicl.2014.07.003 25161896 PMC4141977

[30] Fu Z Tu Y Di X Du Y Pearlson GD Turner JA Characterizing dynamic amplitude of low-frequency fluctuation and its relationship with dynamic functional connectivity: an application to schizophrenia. NeuroImage (2017) 180:1–13. 10.1016/j.neuroimage.2017.09.035 PMC5860934 28939432

[31] Calhoun VD Dynamic connectivity states estimated from resting fMRI identify differences among schizophrenia, bipolar disorder, and healthy control subjects. Front Hum Neurosci (2014) 8:897–13. 10.3389/fnhum.2014.00897 PMC4224100 25426048

[32] Andrews-Hanna JR Smallwood J Spreng RN The default network and self-generated thought: component processes, dynamic control, and clinical relevance. Ann N Y Acad Sci (2014) 1316:29–52. 10.1111/nyas.12360 24502540 PMC4039623

[33] Kennis M van Rooij SJH van den Heuvel MP Kahn RS Geuze E Functional network topology associated with posttraumatic stress disorder in veterans. Neuroimage Clin (2016) 10:302–9. 10.1016/j.nicl.2015.12.008 PMC4724037 26900570

[34] Miller DR Logue MW Wolf EJ Maniates H Robinson ME Hayes JP Posttraumatic stress disorder symptom severity is associated with reduced default mode network connectivity in individuals with elevated genetic risk for psychopathology. Depress Anxiety (2017) 34:632–40. 10.1002/da.22633 PMC5523965 28494120

[35] Patriat R Birn RM Keding TJ Herringa RJ Default-mode network abnormalities in pediatric posttraumatic stress disorder. J Am Acad Child Adolesc Psychiatry (2016) 55:319–27. 10.1016/j.jaac.2016.01.010 PMC4808564 27015723

[36] Liu F Xie B Wang Y Guo W Fouche J-P Long Z Characterization of post-traumatic stress disorder using resting-state fMRI with a multi-level parametric classification approach. Brain Topogr (2014) 28:221–37. 10.1007/s10548-014-0386-2 25078561

[37] Jin C Jia H Lanka P Rangaprakash D Li L Liu T Dynamic brain connectivity is a better predictor of PTSD than static connectivity. Hum Brain Mapp (2017) 38:4479–96. 10.1002/hbm.23676 PMC6866943 28603919

[38] Preti MG Bolton TA Van De Ville D The dynamic functional connectome– state-of-the-art and perspectives. NeuroImage (2016) 160:1–33. 10.1016/j.neuroimage.2016.12.061 28034766

[39] Blake DD Weathers FW Nagy LM Kaloupek DG Gusman FD Charney DS Keane TM The development of a Clinician-Administered PTSD Scale. J Trauma Stress (1995) 8:75–90. 10.1007/bf02105408 7712061

[40] Zung WW A rating instrument for anxiety disorders. Psychosomatics (1971) 12:371–9. 10.1016/S0033-3182(71)71479-0 5172928

[41] Zung WW A Self-Rating Depression Scale. Arch Gen Psychiatry (1965) 12:63–70. 10.1001/archpsyc.1965.01720310065008 14221692

[42] Jenkinson M Bannister P Brady M Smith S Improved optimization for the robust and accurate linear registration and motion correction of brain images. NeuroImage (2002) 17:825–41. 10.1006/nimg.2002.1132 12377157

[43] Yan C-G Craddock RC Zuo X-N Zang Y-F Milham MP Standardizing the intrinsic brain: towards robust measurement of inter-individual variation in 1000 functional connectomes. NeuroImage (2013) 88:1–17. 10.1016/j.neuroimage.2013.04.081 23631983 PMC4074397

[44] Leonardi N Van De Ville D On spurious and real fluctuations of dynamic functional connectivity during rest. NeuroImage (2015) 104:430–6. 10.1016/j.neuroimage.2014.09.007 25234118

[45] Ball T Breckel TPK Mutschler I Aertsen A Schulze-Bonhage A Hennig J Variability of fMRI-response patterns at different spatial observation scales. Hum Brain Mapp (2011) 33:1155–71. 10.1002/hbm.21274 PMC6870263 21404370

[46] Pajula J Tohka J Effects of spatial smoothing on inter-subject correlation based analysis of FMRI. Magn Reson Imaging (2014) 32:1114–24. 10.1016/j.mri.2014.06.001 24970023

[47] Leech R Sharp DJ The role of the posterior cingulate cortex in cognition and disease. Brain (2013) 137:12–32. 10.1093/brain/awt162 23869106 PMC3891440

[48] Guo W Liu F Yao D Jiang J Su Q Zhang Z Decreased default-mode network homogeneity in unaffected siblings of schizophrenia patients at rest. Psychiatry Res Neuroimaging (2014) 224:218–24. 10.1016/j.pscychresns.2014.08.014 25242670

[49] Liu F Guo W Fouche J-P Wang Y Wang W Ding J Multivariate classification of social anxiety disorder using whole brain functional connectivity. Brain Struct Funct (2013) 220:101–15. 10.1007/s00429-013-0641-4 24072164

[50] Buckner RL Andrews-Hanna JR Schacter DL The brain’s default network: anatomy, function, and relevance to disease. Ann N Y Acad Sci (2008) 1124:1–38. 10.1196/annals.1440.011 18400922

[51] Zhang Y Liu F Chen H Li M Duan X Xie B Intranetwork and internetwork functional connectivity alterations in post-traumatic stress disorder. J Affect Disord (2015) 187:114–21. 10.1016/j.jad.2015.08.043 26331685

[52] Cole MW Repovš G Anticevic A The frontoparietal control system. Neuroscientist (2014) 20:652–64. 10.1177/1073858414525995 PMC4162869 24622818

[53] Petersen SE Fox PT Posner MI Mintun M Raichle ME Positron emission tomographic studies of the cortical anatomy of single-word processing. Nature (1988) 331:585–9. 10.1038/331585a0 3277066

[54] Yin Y Li L Jin C Hu X Duan L Eyler LT Abnormal baseline brain activity in posttraumatic stress disorder: a resting-state functional magnetic resonance imaging study. Neurosci Lett (2011) 498:185–9. 10.1016/j.neulet.2011.02.069 21376785

[55] Patel NV Finch CE The glucocorticoid paradox of caloric restriction in slowing brain aging. Neurobiol Aging (2002) 23:707–17. 10.1016/S0197-4580(02)00017-9 12392776

[56] Uddin LQ Salience processing and insular cortical function and dysfunction. Nat Rev Neurosci (2015) 16:55–61. 10.1038/nrn3857 25406711

[57] Menon V Uddin LQ Saliency, switching, attention and control: a network model of insula function. Brain Struct Funct (2010) 214:655–67. 10.1007/s00429-010-0262-0 PMC2899886 20512370

[58] Lei D Li K Li L Chen F Huang X Lui S Disrupted functional brain connectome in patients with posttraumatic stress disorder. Radiology (2015) 276:818–27. 10.1148/radiol.15141700 25848901

[59] Sripada RK King AP Welsh RC Garfinkel SN Wang X Sripada CS Neural dysregulation in posttraumatic stress disorder. Psychosom Med (2012) 74:904–11. 10.1097/PSY.0b013e318273bf33 PMC3498527 23115342

[60] Buckner RL Krienen FM Yeo BTT Opportunities and limitations of intrinsic functional connectivity MRI. Nat Neurosci (2013) 16:832–7. 10.1038/nn.3423 23799476

[61] Hutchison RM Womelsdorf T Allen EA Bandettini PA Calhoun VD Corbetta M Dynamic functional connectivity: promise, issues, and interpretations. NeuroImage (2013) 80:360–78. 10.1016/j.neuroimage.2013.05.079 PMC3807588 23707587

