# Supplementary material for: Altered Local and Large-Scale Dynamic Functional Connectivity Variability in Posttraumatic Stress Disorder: A Resting-State fMRI Study
Source: Front Psychiatry. 2019 Apr 12;10:234. doi: 10.3389/fpsyt.2019.00234 (PMC6474202; doi:10.3389/fpsyt.2019.00234)
Supplement: Supplementary file 1 [file DataSheet_1.docx]

**Supplementary Material**

Supplementary Figure 1. The dReHo/dFC maps were spatially smoothed with 4mm, 6mm, 8mm full width at half maximum (FWHM) Gaussian kernels before the Two-sample t-test. Then, Two-sample t-test, with head motion parameter (mean FD Jenkinson), age, sex as covariates, was performed to test the difference in dReHo maps between PTSD group and Healthy controls at each voxel. Gaussian random field(GRF) theory was used for cluster-level multiple comparison correction (cluster-level significance: *p*<0.05, corrected). It is easy to notice that large smoothing kernel spread the area with more variability in dReHo map.


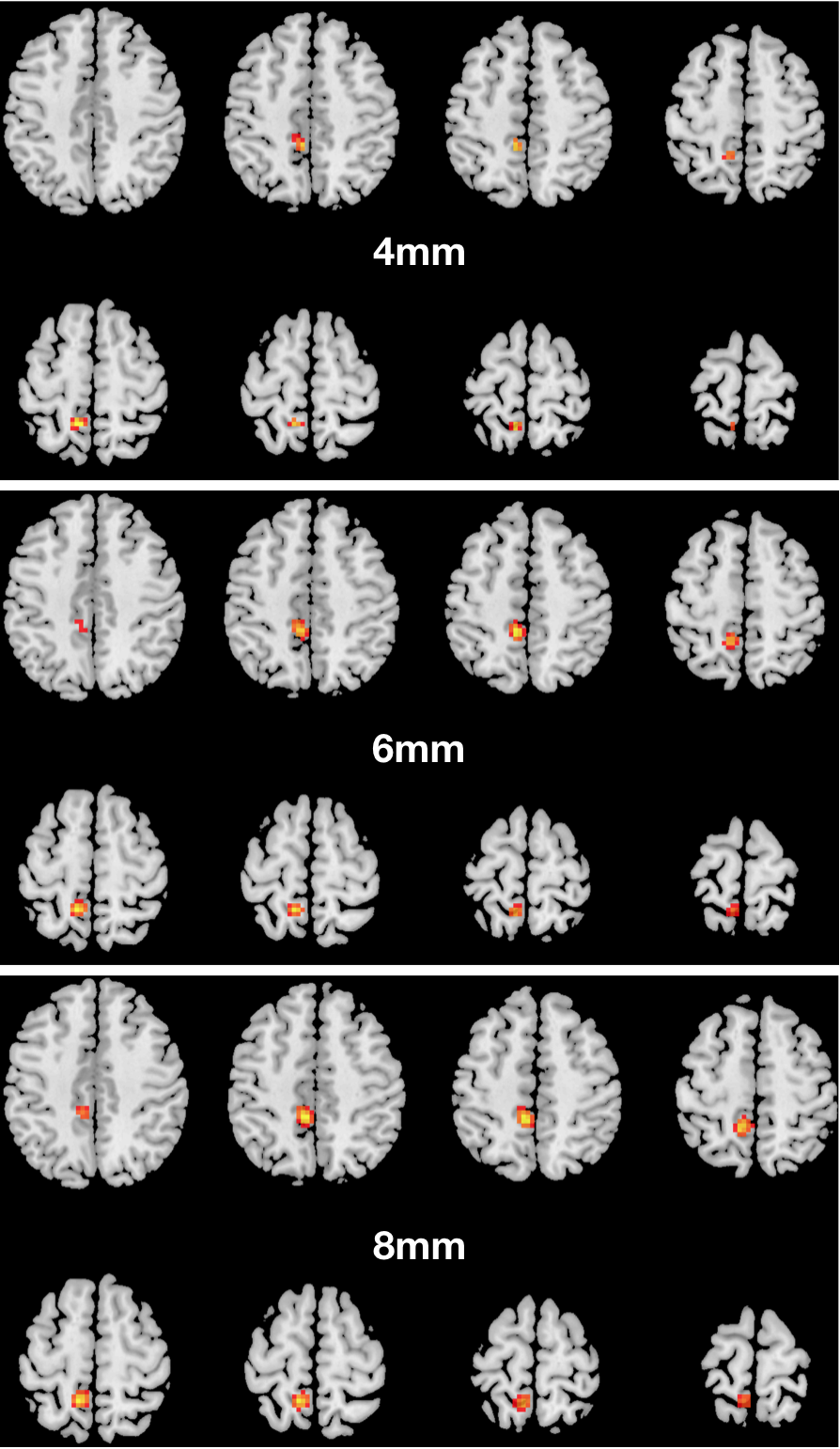


*Supplementary Figure 1.* *The images are from the axial slice of z=44mm to z=72mm anteroposterior axis in MNI coordinate system. The cluster size of left precuneus is 48 voxels while we used 4mm smoothing kernel; 6mm smoothing kernel -104 voxels; 8mm smoothing kernel-122 voxels.*

Supplementary Figure 2. The dFC maps were spatially smoothed with 4mm, 6mm, 8mm full width at half maximum (FWHM) Gaussian kernels in the preprocessing process. Two-sample t-test, with head motion parameter (mean FD Jenkinson), age, sex as covariates, was performed to test the difference in dFC maps between PTSD group and Healthy controls at each voxel. Gaussian random field(GRF) theory was used for cluster-level multiple comparison correction (cluster-level significance: *p*<0.05, corrected). It is easy to notice that large smoothing kernel spread the area with more variability in dFC map.


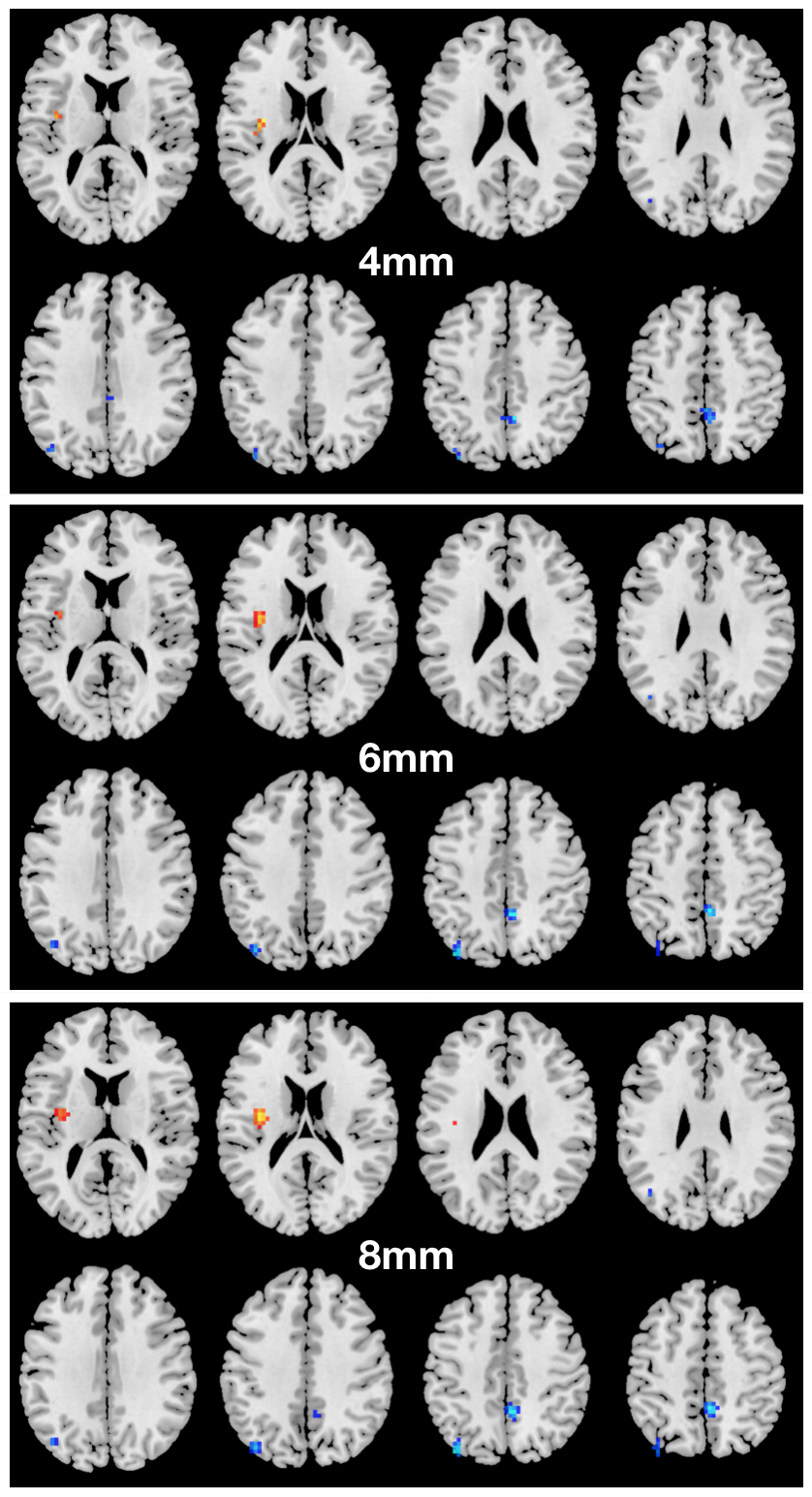


*Supplementary Figure 2. The images are from the axial slice of z=14mm to z=49mm anteroposterior axis in MNI coordinate system. The cluster size of left insula is 13 voxels while we used 4mm smoothing kernel, 6mm smoothing kernel- 33 voxels, 8mm smoothing kernel-45 voxels.*


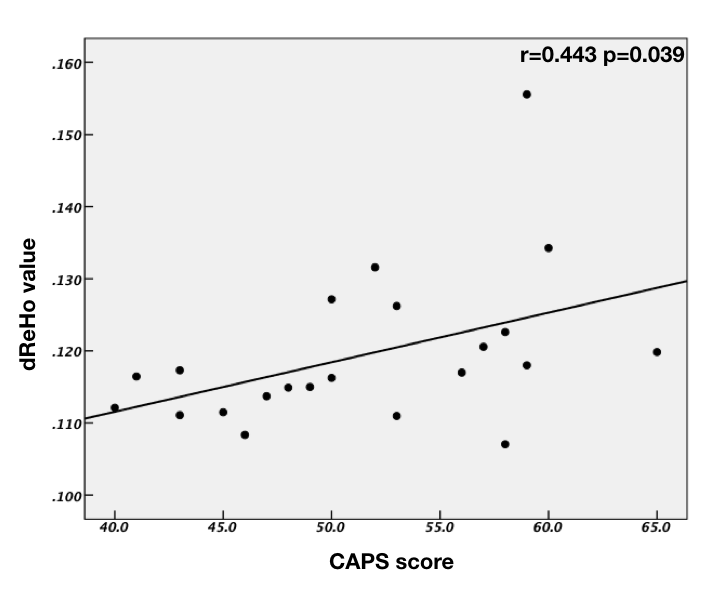

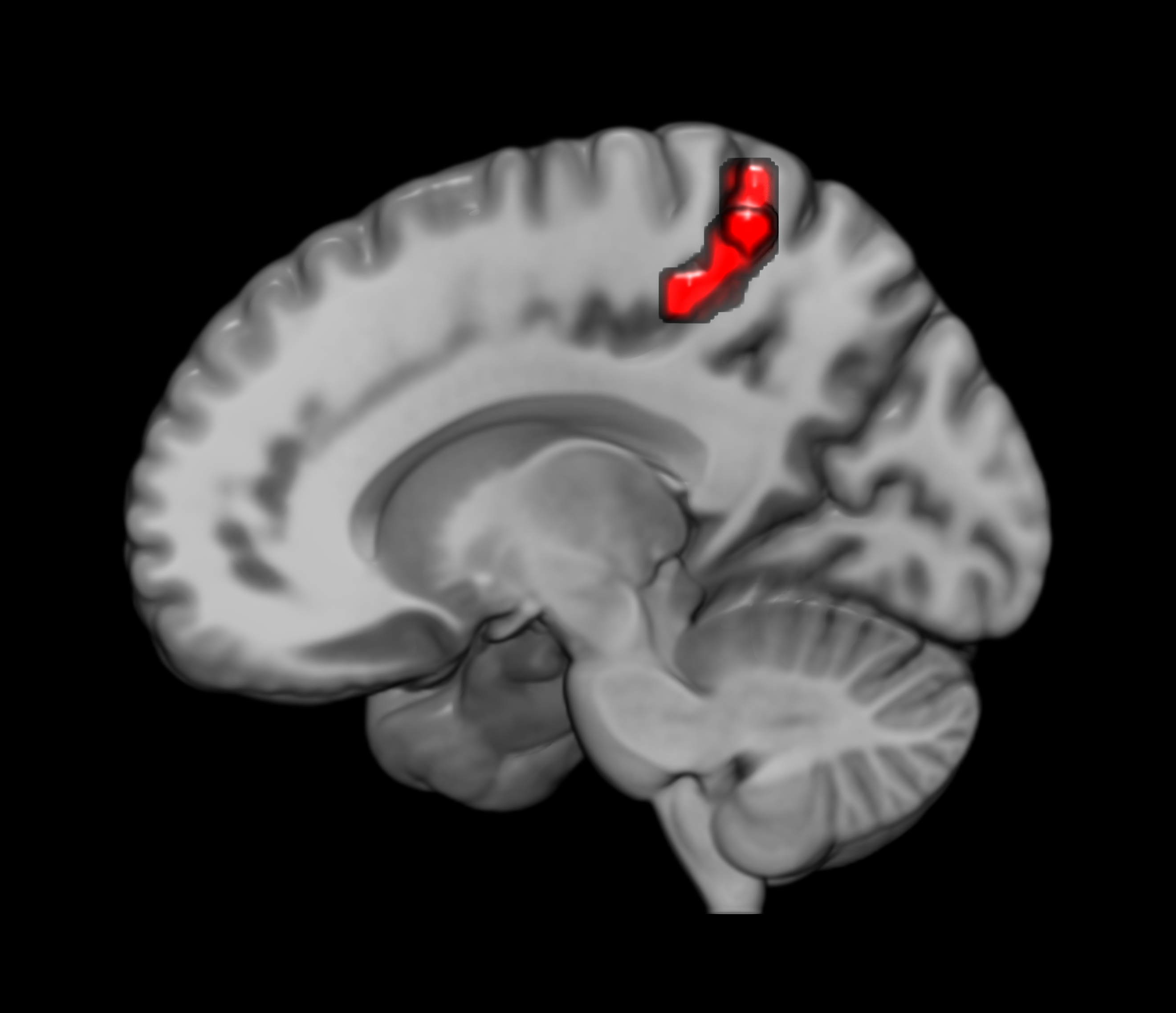


*Supplement figure 3. Correlation between dReHo values and the CAPS scores. The dReHo values of the left PCu was positively correlated with the CAPS scores in PTSD group (r =0.443, p =0.039, uncorrected). The brain slice displays the sagittal view of the left PCu with significant group differences. dReHo, dynamic regional homogeneity; CAPS, Clinical-Administered PTSD Scale; PCu, precuneus.*


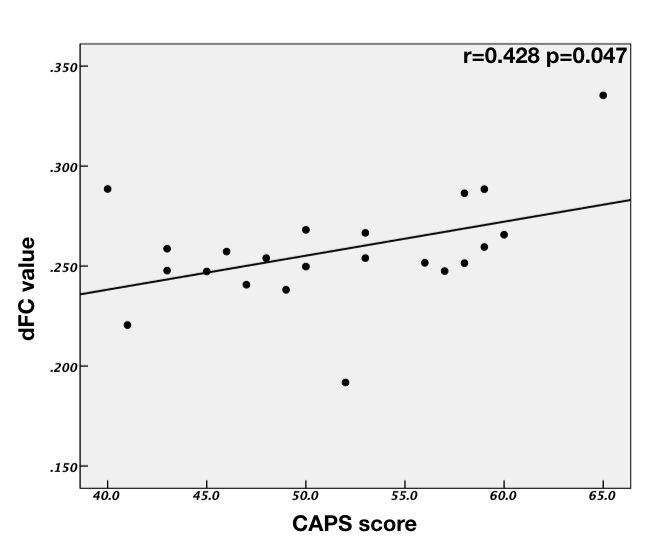

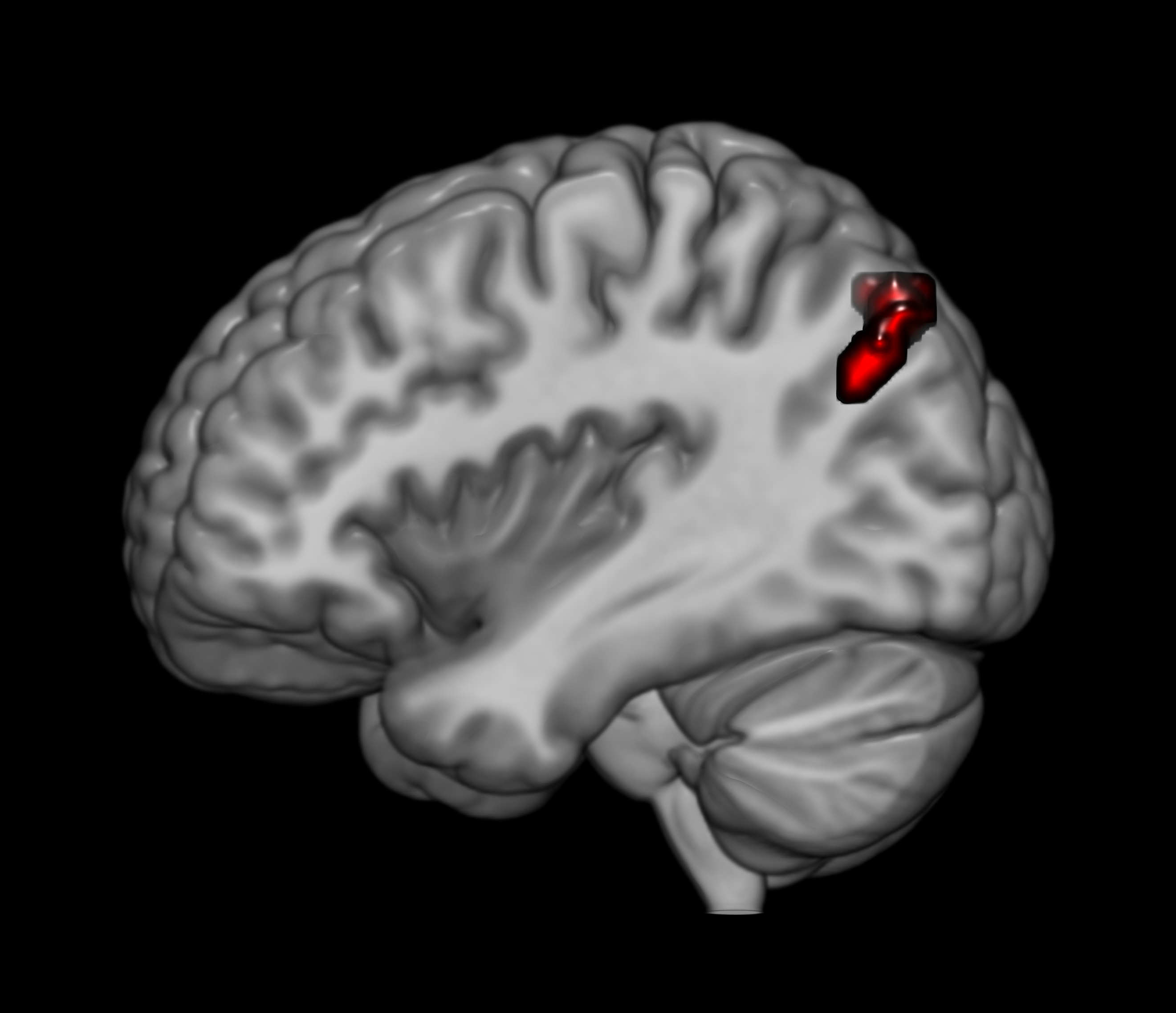


*Supplement figure 4. Correlation between dFC values and the CAPS scores. The pattern of restrained variability between the seed region (left PCu) and the left IPL was positively correlated with the CAPS scores in PTSD group (r =0.428, p =0.047, uncorrected). The brain slice displays the sagittal view of the left IPL with significant group differences. dReHo, dynamic regional homogeneity; CAPS, Clinical-Administered PTSD Scale; PCu, precuneus; IPL, inferior parietal lobe.*
